# Enriching leukapheresis improves T cell activation and transduction efficiency during CAR T processing

**DOI:** 10.1016/j.omtm.2021.02.002

**Published:** 2021-02-06

**Authors:** Elsa Noaks, Carlotta Peticone, Ekaterini Kotsopoulou, Daniel G. Bracewell

**Affiliations:** 1 Department of Biochemical Engineering, University College London, Bernard Katz Building, Gower Street, London WC1E 6BT, UK; 2 Autolus Therapeutics Ltd., The Mediaworks, 191 Wood Lane, White City, London W12 7FP, UK

**Keywords:** CAR T-cell, cell therapy, manufacture, enrichment, monocytes, activation, transduction, leukapheresis

## Abstract

The majority of CD19-directed CAR T cell products are manufactured using an autologous process. Although using a patient's leukapheresis reduces the risks of rejection, it introduces variability in starting material composition and the presence of cell populations that might negatively affect production of chimeric antigen receptor (CAR) T cells, such as myeloid cells. In this work, the effect of monocytes (CD14) on the level of activation, growth, and transduction efficiency was monitored across well plate and culture bag platforms using healthy donor leukapheresis. Removal of monocytes from leukapheresis improved the level of activation 2-fold, achieving the same level of activation as when initiating the process with a purified T cell starting material. Two activation reagents were tested in well plate cultures, revealing differing sensitivities to starting material composition. Monocyte depletion in culture bag systems had a significant effect on transduction efficiency, improving consistency and increasing the level of CAR expression by up to 64% compared to unsorted leukapheresis. Cytotoxicity assays revealed that CAR T cell products produced from donor material depleted of monocytes and isolated T cells consistently outperformed those made from unsorted leukapheresis. Analysis of memory phenotypes and gene expression indicated that CAR T cells produced using depleted starting material displayed a more rested and naive state.

## Graphical Abstract

**Figure:**
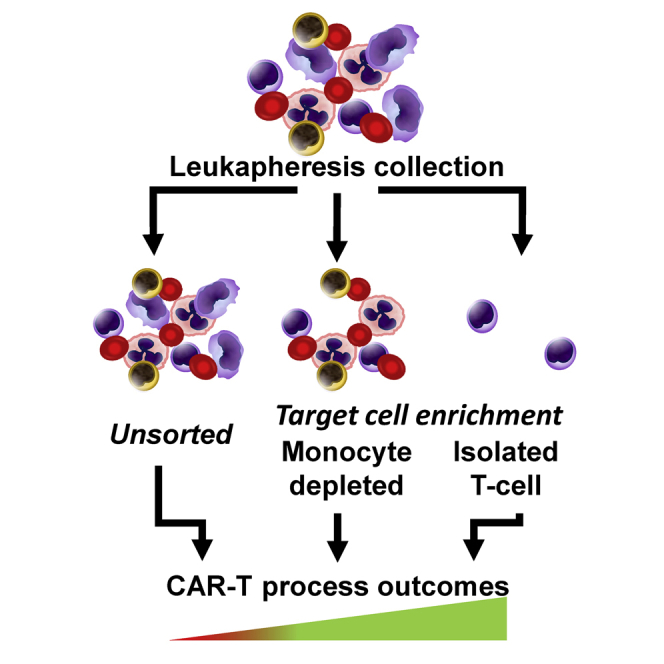

## Teaser

The success of CAR T cell manufacturing and final product function is influenced by the composition of the donor starting material. In this work, we show that upstream depletion of specific cell populations can enhance processing outcomes such as activation, transduction, and phenotype of the therapeutic product.

## Introduction

**Figure 1:**
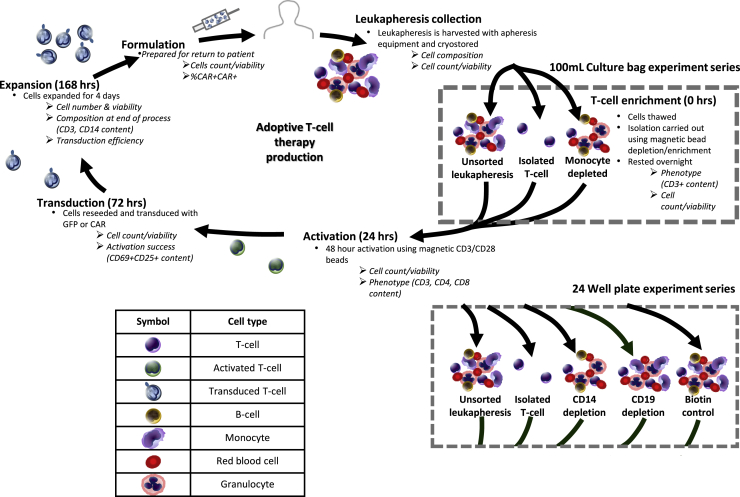
Summary of process stages in CAR T cell manufacture and experiment structure used in this study, carried out in a 100-mL culture bag and 24-well plate Round bullet points describe the processes used in the study, and italic text denotes variables measured. A key is provided for cell illustrations. A timescale is included for the different processing stages.

Chimeric antigen receptor (CAR) T cell therapies have achieved unparalleled success in treatment of refractory blood cancers.^1^, ^2^, ^3^, ^4^, ^5^ The first effective and persistent CAR T cell therapy was developed in 2003, killing leukemia cells in a murine model.^6^ Within 15 years, two products were approved for use in humans with refractory cancer, Yescarta (non-Hodgkin lymphoma, Kite Pharma) and Kymriah (B cell acute lymphoblastic leukemia, Novartis), and over 700 more CAR T cell products are in clinical trials.^7^^,^^8^ Although there has been rapid translation from conception to commercialized therapy and development of processing technologies, manufacture of CAR T cell products has continued to follow a rigid structure that has remained relatively unchanged since their conception. A conventional pathway for CAR T cell manufacture is shown in Figure 1.

The majority of CAR T cell manufacture depends on collection of starting material directly from the patient , with 93% of CD19-directed CAR T cells being autologous.^9^ Starting material is typically obtained by leukapheresis, which yields a white blood cell (WBC)-rich blood fraction. T cells are then activated by providing signals that trigger T cell division before being transduced with a viral vector to express the CAR sequence. Cells are then expanded to achieve the required dose size before being formulated, cryopreserved, and administered to the individual. The composition and quality of leukapheresis products can vary widely from donor to donor, especially in individuals with refractory cancer.

The variability in starting material can make it challenging to establish a reproducible CAR T cell manufacturing platform, resulting in failure to meet dose requirements or, in extreme cases, cause resistance to CAR T cells.^10^ To overcome this issue, some groups purify leukapheresis products to achieve a more homogeneous starting material consisting of mainly CD3^+^ T cells.^4^^,^^11^^,^^12^ Certain cell populations shown to be disruptive to key processing outcomes, such as natural killer (NK) cells (CD56^+^)^13^^,^^14^ and monocyte cells (CD14^+^).^15^ Monocytes are adherent^16^^,^^17^ and possess the capacity to attach to manufacturing surfaces, such as CD3/CD28 activation beads,^15^ and have a detrimental effect on production of CAR T cells.^18^^,^^19^ In batches where donors present high levels of CD14^+^ cells and have failed to pass requirements for further processing, depletion of monocytes is often attempted as a salvage operation for the remaining donor material before reattempting CAR T cell production.^18^^,^^20^ B cells (CD19^+^) are another potential contaminant in starting material from individuals with leukemia because of the risk associated with transducing cancerous cells as part of the manufacturing process.^10^

This study explores how the composition of an individual’s leukapheresis material can influence the key stages of the process: activation, transduction, and expansion. Healthy donor leukapheresis products were purified by depletion of specific cell populations, monocytes or B cells, and activated using TransAct or Dynabeads. Two different retroviral vectors were used for transduction, resulting in T cells that express a green fluorescent protein (GFP) or FMC63,^21^ a CAR structure containing an anti-CD19 single-chain variable fragment (scFv) that targets B lymphocytes. The resulting CAR T cell products were compared based on their target-mediated cytotoxicity, cytokine secretion, and gene expression to understand the implications of the initial leukapheresis composition for product characteristics and functionality.

## Results

### T cell enrichment of donor leukapheresis material

To explore the effect of starting material composition on the outcome of CAR T cell processing, leukaphereses from healthy donors were enriched using magnetic cell sorting kits, producing populations depleted of monocytes (CD14), B cells (CD19), or all blood cells (CD14, CD15, CD16, CD19, CD34, CD36, CD56, CD123, and CD235a) to produce untouched T cells (isolated T cells) and a non-depleted control. The results from these sorting processes are summarized in Table 1, including ranges for the leukapheresis donors used in this study. In monocyte-depleted cultures, CD14^+^ cells underwent at least a 10-fold reduction, from 9.3%–32% to 0.1%–1.1%, and, as a result of partial enrichment, CD3^+^ cells increased by ∼10%. A similar outcome was seen in the CD19-depleted populations, where, after depletion, CD19^+^ cells dropped from 7.8%–13% to 0.05%–0.13%. For most donors, cultures in which all cells were depleted apart from T cells were more than 95% CD3^+^, with all other WBC populations falling below 2% each. There was an exception to this, with one donor only producing an 81.3% CD3^+^ population, thought to be caused by residual bead-bound CD56/CD14/CD19 cells in the isolated T cell harvest. An unsorted leukapheresis was processed alongside depleted starting materials as a control. A second control was produced by replicating the CD14 depletion protocol using biotin-conjugated beads without antibody targeting, producing a starting material similar in composition to the unsorted leukapheresis (biotin control). The majority of tested donors fell within literature ranges for peripheral blood mononuclear cell (PBMC), whole blood or apheresis .^22^ Because granulocytes were not retained after cryopreservation, with CD16^+^ cells accounting for 3% or less of the population, they were not monitored in this study.

**Table 1:** CD markers measured in flow cytometry to identify cell phenotype, activation, and transduction levels

Marker	Purpose	Literature values (%)	Donor range (%)	Purified condition ranges (%)			
				Isolated CD3 T cells	CD14 depletion	CD19 depletion	Biotin control
CD235a	Expressed by red blood cells	–	2.2–15	0.07–2.4	2.9–19	2.4–16	2.8–6.6
CD45	Expressed by all WBCs	–	83–96	98–99	79–95	82–97	85–97
CD3	Pan T cell marker, expressed by all T cells	44–84^22^	25–63	81–97	57–75	28–72	25–64
CD4	A subset of CD3+ T cells, T helper cells	23–59^22^	53–81	53–81	53–81	53–81	53–81
CD8	A subset of CD3+ T cells, cytotoxic T cells	10–43^22^	13–35	13–35	13–35	13–35	13–35
CD14	Expressed by monocytes	8.0–45^23^	9.3–32	0.1–0.7	0.1–1.1	12–28	12–27
CD19	Expressed by B cells	4.4–28^22^	2.3–13	0.3–1.3	5.3–18	0.05–0.13	2.1–15
CD56	Natural killer (NK) cells and a subset of CD3^+^ T cells (NK T cells)	3.7– 4.6^22^	2.6–8.9	0.6–1.5	3.6–8.5	3.7–6.2	3.5–5.7

### Monocytes influence activation success in 24-well plates

**Figure 2:**
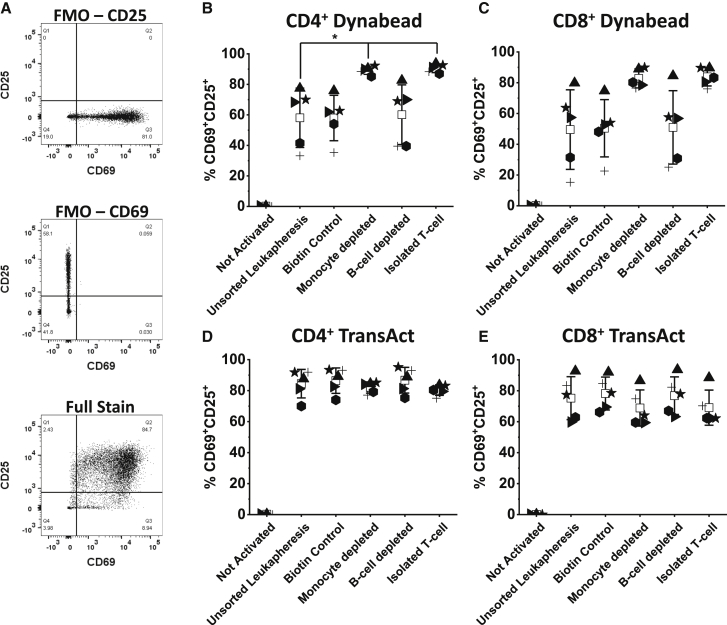
Expression of the activation markers CD69^+^ and CD25^+^ by CD4^+^ and CD8^+^ populations after Dynabead or TransAct activation in a 24-well plate CD69 and CD25 expression by T cells after 48-h activation stimulated by Dynabeads or TransAct, was measured using flow cytometry for donor 4 (▲), donor 5 (▸), donor 6 (), donor 7 (+), and donor 8 (★). An example of the flow cytometry gating is shown in (A) Fluorescence minus one (FMO) controls taken from donors 5/6 and full stain is the result from donor 5 isolated T cell TransAct cultures. CD69 and CD25 expression was measured for all starting material conditions (unsorted leukapheresis, biotin control, monocyte depletion, B cell depletion, and isolated T cells) where (B) CD4^+^ Dynabeads, (C) CD8^+^ Dynabeads, (D) CD4^+^ TransAct, and (E) CD8^+^ TransAct. A “not activated” control was included for comparison. In all cases, n = 5; error bars represent the mean ± 1 SD. The mean of the value is represented by □. Statistical significance was determined by one-way ANOVA and Dunnett’s hypothesis testing; ∗p < 0.05 and ∗∗p < 0.01 compared with unsorted leukapheresis products.

Activation is crucial for manufacture of CAR products because it triggers cell division, facilitating retroviral transduction. One of the most widely used platforms for T cell activation is CD3/CD28 antibody-conjugated paramagnetic microbeads, such as Dynabeads.^9^^,^^11^^,^^24^^,^^25^ In this study, Dynabeads and TransAct (a CD3/CD28 polymeric nanomatrix) were used as activation reagents. All experiments were processed via a conventional CAR T cell manufacturing cycle in 24-well plates, as outlined in Figure 1. TransAct and Dynabeads were added, supplemented with 300 IU/mL of interleukin-2 (IL-2), and cultured for 48 h. Activation was measured by comparing the simultaneous expression of the activation markers CD25^+^ and CD69^+^ in CD4 and CD8 T cell populations (Figures 2A–2E).

Because of the disruptive nature of CD14^+^ monocytes, it was expected that their removal would result in enhanced activation compared to unsorted leukapheresis, potentially approaching the performance of a homogeneous CD3^+^ starting material. In CD3/CD28 Dynabead-activated cultures (Figures 2B and 2C), there was greater expression of key activation markers across all donors upon monocyte cell removal and T cell enrichment. Donor 6 experienced the greatest increase in activation level, with a 2-fold (41.5% to 85.2%) and 2.5-fold (31.4% to 80.2%) increase in CD69^+^CD25^+^ expression in CD4^+^ and CD8^+^ T cells, respectively, compared to unsorted leukapheresis. To confirm that the improvement in activation was a result of CD14 depletion, two additional conditions were tested. The CD19^+^ B cell population in the tested donors accounted for a similar proportion (10.8% ± 4%) of the WBC population as CD14^+^ cells (14.2% ± 6.3%). If the increase in activation was a result of partial T cell enrichment because of CD14 removal, then CD19-depleted cultures should not produce a similar result. An additional biotin-depleted control was included to establish whether the process of depleting the cells had any effect on processing outputs rather than the specific enrichment itself. Neither CD19 depletion nor biotin control showed any improvement in expression of CD69^+^/CD25^+^ in T cells or consistency of activation between donors compared with unsorted leukapheresis. On the other hand, there was an increase in the level of activation in CD14-depleted (p < 0.05) and isolated CD3^+^ population cultures versus the biotin control. TransAct-activated cultures performed differently than those processed with Dynabeads (Figures 2D and 2E). For CD4^+^CD69^+^CD25^+^ populations, removal of CD14^+^ cells from culture and isolation of CD3^+^ T cells slightly reduced the variability between donors; however, this pattern is absent in CD8^+^CD69^+^CD25^+^ populations, and there is no overall trend for increased levels of activation by pre-sorting cells.

**Figure 3:**
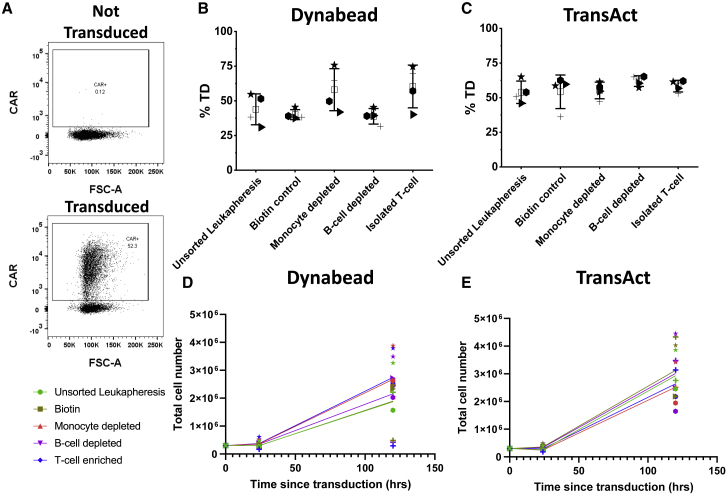
CAR retroviral transduction efficiency and growth curves for both activation reagents and tested conditions tested in a 24-well plate for donor 5 (▸), donor 6 (), donor 7 (+), and donor 8 (★) (A) Example of flow cytometry gating for transduction efficiency (%TD). (B and C) (B) Transduction efficiency achieved after Dynabead activation and (C) transduction efficiency achieved after TransAct activation. n = 4, error bars represent the mean ± 1 SD. The mean of the value is represented by □. (D and E) Growth curves start after transduction reseeding (where 0 h since transduction is equivalent to 48 h after activation) for (D) Dynabead-activated and (E) TransAct-activated cultures.

Retroviral transduction using the FMC63 vector, modified to contain the marker epitope RQR8,^26^ showed that transduction efficiency was only improved by monocyte depletion and T cell isolation in certain donors (Figures 3B and 3C) when activating with Dynabeads. The growth of cell populations was recorded throughout the process; growth curves were produced for expansion after transduction reseeding (day 3) to the end of expansion (day 8), based on total viable cell counts. Overall, the composition of the starting material did not alter the level of cell expansion, with final product composition always consisting of more than 97% T cells. One exception to this was donor 7 Dynabead cultures, displaying heavily reduced expansion in unsorted leukapheresis, B cell-depleted and biotin control derived material, achieving a final cell population of 2.94–4.85 × 10^5^, whereas monocyte-depleted and isolated T cells behaved in line with the other tested donors, yielding 2.46 and 2.21 × 10^6^ cells, respectively. Donor 7 possessed one of the largest CD14 populations of 32.2%, up to 3-fold higher than the other donors, potentially causing the suppressed growth. Generally, TransAct achieved higher expansion compared with Dynabeads (Figures 3D and 3E). Further studies were performed at a larger scale in cell culture bags and under more representative clinical manufacturing conditions to confirm and examine the effects of starting material composition on process output.

### Scaling into 100-mL culture bags confirms the influence of monocytes on activation

**Figure 4:**
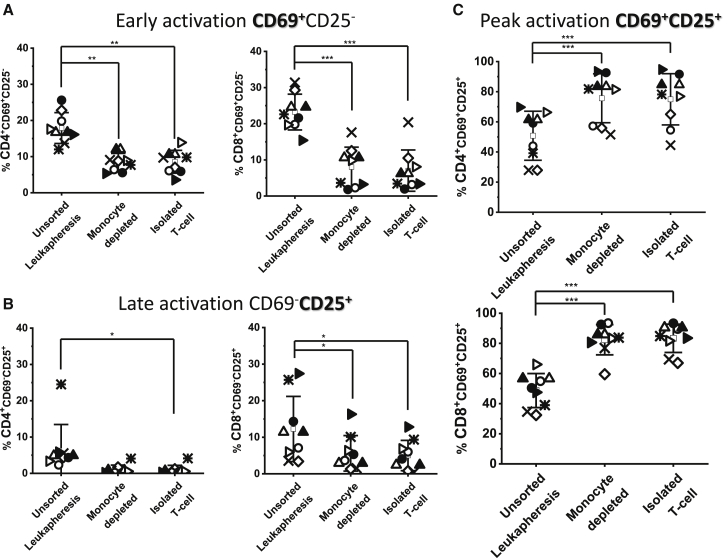
Review of activation marker CD69 and CD25 expression by CD4^+^ and CD8+ T cell populations after CD3/CD28 bead-based activation in a 100-mL culture bag (A) Early activation (CD69^+^CD25^−^), (B) late activation (CD69^−^CD25^+^), and (C) peak activation (CD69^+^CD25^+^) by CD4^+^ and CD8^+^ T cells after 48-h Dynabead activation of unsorted leukapheresis, monocyte-depleted, and isolated T cell starting populations. 5 different donors were activated: donor 1 (◇), donor 2 (○), donor 3 (⨯), donor 4 (▵), and donor 5 (▹). Donors 2, 4, and 5 were replicated for subsequent CAR viral transduction alongside donor 9 (denoted donor 2_CAR [●], donor 4_CAR ▴], donor 5_CAR [▸], and donor 9_CAR [★]). Error bars represent the mean ± 1 SD, where the mean of the value is represented by □. Statistical significance was determined by one-way ANOVA and Dunnett’s hypothesis testing; ∗p < 0.05, ∗∗p < 0.01, and ∗∗∗p < 0.001.

In culture bag studies, activation was carried out using CD3/CD28 Dynabeads supplemented with 300 IU/mL of IL-2. Because they did not affect activation and transduction, CD19-depleted and biotin control conditions were not carried forward (Figures 2A–2E). TransAct was tested in culture bags; however, as seen in well plates, the levels of activation, transduction, and expansion were consistent (Figure S1) across all tested conditions and were not examined further. Cells from the different experimental conditions were seeded into 100-mL culture bags and rested overnight before starting activation and subsequent transduction and expansion phases. As with the small-scale studies, activation success was measured 48 h after addition of IL-2 and CD3/CD28 Dynabead stimulation by monitoring the levels of the activation markers CD69^+^ and CD25^+^ in CD4 and CD8 T cell populations (Figure 4C). Donors were transduced using two different retroviral vectors, resulting in expression of GFP (n = 5) or the FMC63 CAR (n = 4). For all donors, the level of activation was significantly higher in CD14-depleted (p ≤ 0.001) or isolated T cell (p ≤ 0.001) cultures. On average, 53% and 78% increases in CD25^+^CD69^+^ expression in the CD4 and CD8 T cell populations, respectively, were observed compared to unsorted leukapheresis.

**Figure 5:**
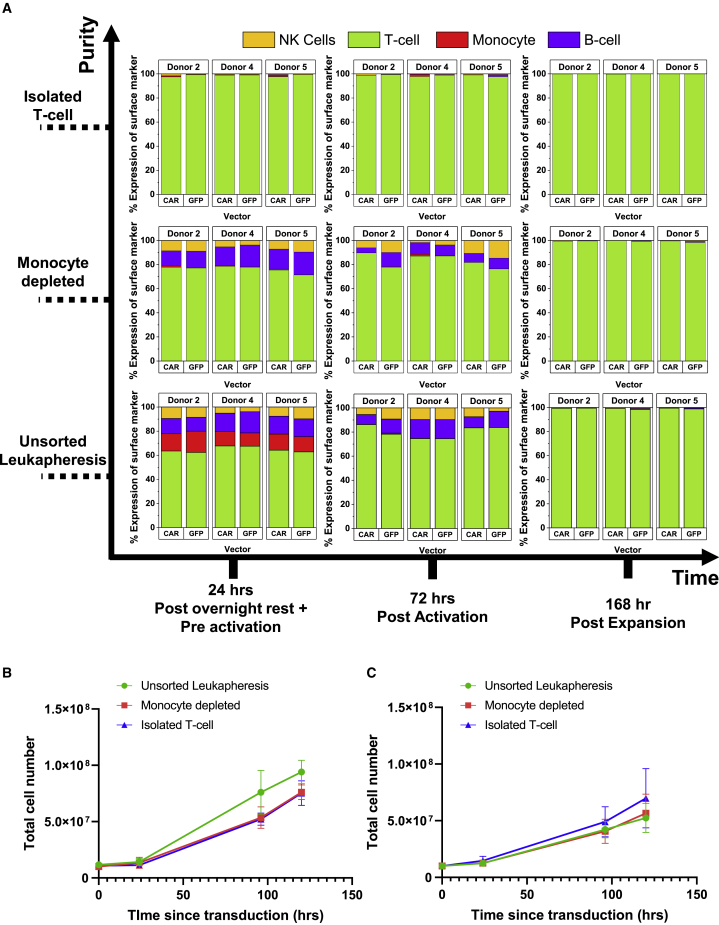
Change in cell populations before/after key process stages and growth curves for GFP/CAR cultures in a 100-mL culture bag study (A) Change in material composition over the experimental period for T cell, monocyte, B cell, and NK cell populations. (B and C) Growth curves for (B) GFP and (C) CAR after transduction (where 0 h since transduction is equivalent to 48 h after activation); n = 4 (CAR) and n = 5 (GFP). Error bars represent the mean ± 1 SD.

Cell expansion was recorded by regular cell counts, and composition of material was monitored (CD3, CD14, CD56, and CD19) at key processing points: pre-activation, post-activation/pre-transduction, and upon completion of expansion (Figure 5A). In unsorted leukapheresis, the CD14 population was absent 48 h after activation; this was likely caused by medium selection^27^ and loss to surfaces when transferring between culture bags. The CD19 and CD56 populations were still observed in culture after activation but absent from the final product, with persistence attributed to IL-2 supplementation.^28^^,^^29^ Cultures derived from an isolated T cell starting material remained pure throughout the cycle. Although there are distinct differences in starting material prior to activation, by the end of expansion, the population is homogeneous, with only T cells present (>98% CD3^+^). This was observed for the CAR and GFP transduction protocols.

Under all conditions, within the first 72 h of processing (activation and transduction), there was a dramatic reduction in the total cell population (data not shown), with a decrease of up to 54% ± 16.5%. This was caused by cell loss across all populations; in unsorted leukapheresis, on average, there were 38%, 99%, 57%, and 39% reductions in CD3, CD14, CD19, and CD56, respectively. In CD14-depleted material, there was an average 79% decrease in B cells in addition to 52% and 59% reductions in T cells and NK cells. Isolated T cell material also experienced an average loss of 50% of its population. After transduction, the cell population recovered; the growth curves for cultures after reseeding to the end of processing are shown in Figures 5B and 5C. In CAR and GFP cultures, starting material composition did not significantly affect the absolute cell population size at the end of processing.

### Transduction efficiency and the resultant T cell cytotoxicity are improved by monocyte depletion

**Figure 6:**
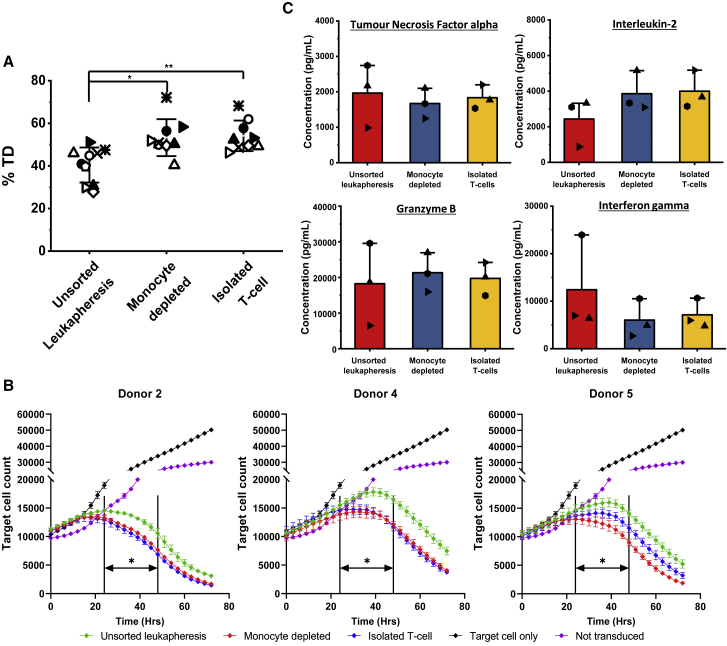
Efficiency of GFP/CAR retroviral transduction, cytotoxicity of CAR+ T cell products, and cytokine profiles (A) Transduction efficiency (% TD) for donor 1 (◇)), donor 2 (○), donor 3 (⨯), donor 4 (▵), and donor 5 (▷), and CAR-transduced donor 2 (●), donor 4 (▴), donor 5 (▸), and donor 9 (★). (B) Target cell mediated cytotoxicity of CAR-transduced material for donors 2, 4, and 5 produced from starting material of unsorted leukapheresis, isolated T-cells and depleted of monocytes. The non-transduced and target cell only condition were included as controls. (C) Expression of cytokines at the end of a Raji cell-based cytotoxicity assay: tumor necrosis factor alpha (TNF-α), interleukin-2 (IL-2), granzyme B (GRANB), and interferon γ (INFγ). Error bars represent the mean ± 1 SD, where the mean of the value is represented by □. Statistical significance was determined by one-way ANOVA and Dunnett’s hypothesis testing; ∗p < 0.05 and ∗∗p < 0.01

Activated cells were transduced with GFP (n = 5) or CAR (FMC63, n = 4) (Figure 6A). CD3-enriched and CD14-depleted cultures consistently achieved higher and more reproducible levels of transduction compared to unsorted leukapheresis (p < 0.01), whereas CD14-depleted and CD3-isolated cultures performed similarly, following the pattern seen in the activation data. As well as the effects of starting material on production of CAR T cells, the potency of the resulting CAR product was also of interest. This was tested by measuring the cytotoxicity of T cells that were transduced with the CAR vector against a CD19^+^ cell line (Figure 6B). The outcomes of these tests repeatedly show that CAR+ T cells produced from starting material depleted of monocytes or from isolated T cells outperform those from unsorted leukapheresis . A faster killing rate was observed in biological products obtained under enriched conditions, resulting in significantly fewer (p < 0.05) target cells remaining in culture between 24 and 48 h than unsorted leukapheresis. Donor 5 has one of the clearest differences between the unsorted leukapheresis and purified fractions but very similar levels of transduction under all conditions. The levels of cytokines associated with cytotoxicity and immune cell modulation—granzyme B (GRANB), interferon gamma (IFNγ), IL2 and tumor necrosis factor alpha (TNF-α)—were measured at the end of a subsequent 48-h Raji cell cytotoxicity assay (Figure 6C) and revealed no changes in expression between the different starting material conditions.

### Understanding the origin of the improved T cell cytotoxicity

**Figure 7:**
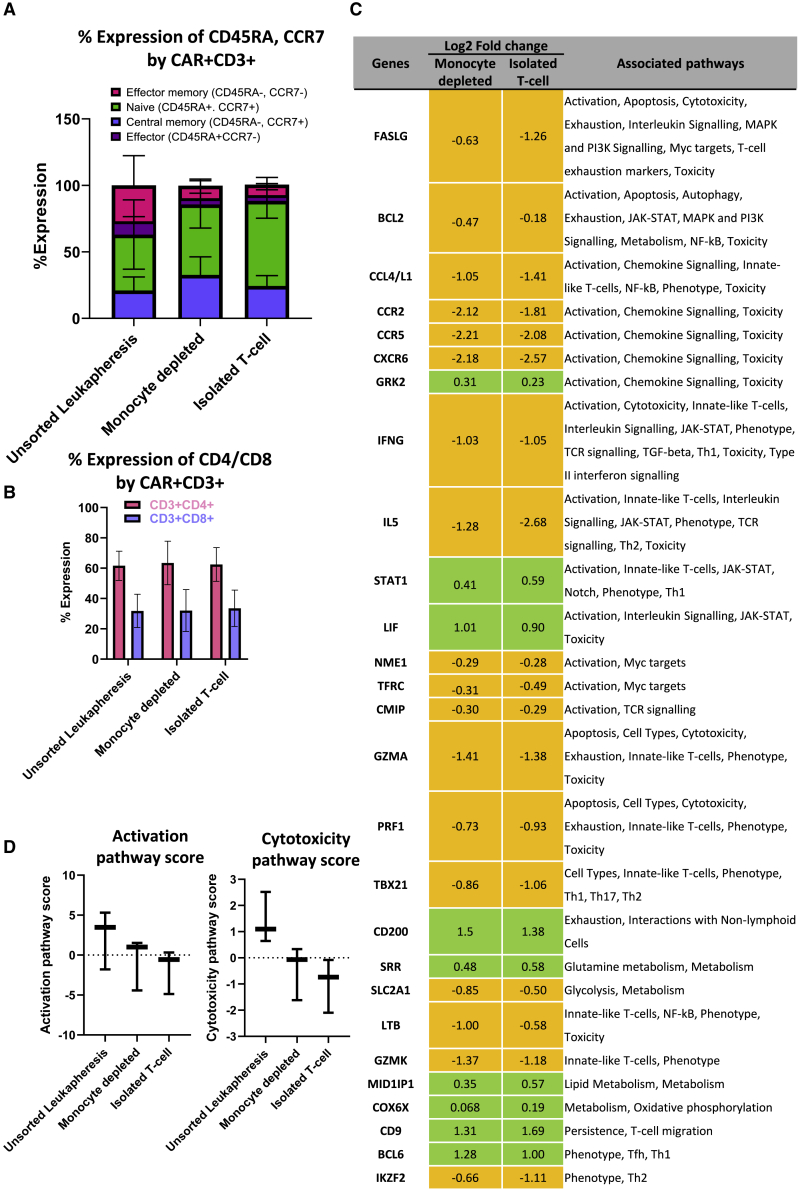
Overview of CAR T cell product memory phenotypes, CD4/CD8 ratios, and gene expression for CAR-transduced donors (A) Review of memory phenotypes after expansion of CAR^+^ T-cells, measured by flow cytometry: CD45RA^+^CCR7^+^ (naive T [T_N_] cells), CD45RA^−^CCR7^+^ (central memory T [T_CM_] cells), and CD45RA^−^CCR7^−^ (effector memory T [T_EM_] cells). (B) Ratio of CD4/CD8 T cells in final CAR T cell products. Error bars represent the mean ± 1 SD. (C) Differences in gene expression of the CAR T cell products at the end of production. Fold changes represent differences compared with unsorted leukapheresis products. Only the top 20 genes that vary under both processed conditions are shown for each condition. (D) Pathway scores for the activation and cytotoxicity gene set from the Nanostring data analysis. n = 3; the bar represents the median value and error bars the minimum and maximum values.

Samples cryopreserved after culture bag expansion were thawed and stained for key memory markers, including CD45RA and CCR7, to identify key T cell populations: central memory T (T_CM_) cells (CD45RA^−^CCR7^+^), effector memory T (T_EM_) cells (CD45RA^−^CCR7^−^), effector T (T_E_) cells (CD45RA^+^CCR7^−^), and naive T (T_N_) cells (CD45RA^+^CCR7^+^) (Figure 7A). Although not significant because of donor variation, the product made from isolated T cells contained the highest proportion of T_N_ cells (34%–79%), with both depleted starting materials yielding larger T_N_ cell populations compared to the unsorted leukapheresis (17%–69%). The CAR T product made from unsorted leukapheresis material possessed a greater population of T_EM_ cells (7.6%–51%), with isolated T cell and monocyte-depleted CAR T products containing up to 50% less. CAR T cells manufactured from monocyte-depleted starting material have the highest levels of T_CM_ cells (p < 0.05). In combination with memory data, RNA was isolated from CAR T cells and used to screen for over 700 genes using a CAR-T Characterization Panel and Nanostring nCounter Sprint cartridge (Figure 7D). The analysis of RNA data focused on comparing CAR T-cells derived from depleted starting materials to those from unsorted leukapheresis , and the data shown summarizes the top 20 genes (according to significance, adjusted p value) for the two conditions. Of the 27 displayed genes, 14 are associated with activation, with 70% of them being downregulated compared with the unsorted leukapheresis CAR T products. Because of the small sample size of this study, the gene expression data had a false discovery rate of 1. The Nanostring analysis also produces pathway scores, which summarize changes in the expression level of biologically related gene groups. The activation and cytotoxicity pathways confirm (Figure 7D) the upregulation of activation- and cytotoxicity-associated genes in CAR T cells derived from unsorted leukapheresis compared with monocyte-depleted and isolated T cell material.

## Discussion

Monocytes can be disruptive to traditional CAR T cell production processes,^18^^,^^20^ inhibiting T cell expansion,^4^^,^^18^^,^^23^ activation, and transduction.^4^^,^^20^ To overcome this issue, some groups have implemented methods to reduce the CD14 content in starting material used for immunotherapy, such as elutriation^15^^,^^23^ or isolation of T cells from starting material.^30^ Such methods do not always achieve complete removal of these populations,^11^^,^^30^ and not all CAR T cell manufacturing cycles adopt additional purification stages to isolate populations,^1^^,^^18^ so the effect of these cells in starting material on process outcomes and product quality is still of interest. In this study, we demonstrated and explored the extent of CD14^+^ cell-related effects on key processing steps and product quality across two culture scales (24-well plates and 100-mL bags) and activation reagents.

When stimulating cultures with CD3/CD28 Dynabeads, removal of monocytes and complete isolation of T cells enhanced the level, consistency, and reproducibility of activation compared with processes started with unsorted leukapheresis. This pattern was present in both culture scales but was more evident in the 100-mL differentiation bag geometry. Conversely, TransAct-stimulated cultures were not sensitive to starting material composition, with all conditions producing nearly identical levels of CD25^+^CD69^+^ expression. Dynabeads and TransAct rely on presentation of the costimulatory signals CD3/CD28 to trigger T cell activation. Dynabeads are paramagnetic beads with a diameter of ∼4.5 μm, whereas TransAct is a polymeric nanomatrix with a diameter of ∼100 nm.^31^ Monocytes are large (10–18 μm) adherent WBCs with the ability to engulf foreign material.^16^ It is likely that, in unsorted leukapheresis, biotin controls, or CD19-depleted cultures, where activation is suppressed, monocytes are attaching to or engulfing Dynabeads,^32^ reducing the number of sites available for activation to take place. The smaller diameter of TransAct may make it more difficult for monocytes to adhere to while also providing a greater surface area for activation to take place. Adding more Dynabeads to the culture could improve activation success but may lead to higher levels of cell apoptosis^33^ and reduced T cell expansion^34^ while increasing the difficulty of bead removal at the end of CAR manufacture.

Following 48-h activation, cells were transduced with a gamma-retrovirus (γ-retrovirus) encoding a GFP or FMC63 sequence. γ-Retroviral transduction can only occur in mitotic cells as the virus can only integrate into dividing cells.^35^ Because T cells can only begin to divide after they are activated,^36^ it was expected that greater levels of activation would increase transduction. In 24-well plate experiments, purification of leukapheresis only improved the transduction efficiency in two of the four donors tested and only when activating with Dynabeads. It was expected that, due to their greater level of activation, all samples depleted of CD14 and isolated for T cells should have demonstrated a higher and more consistent level of transduction. When scaling Dynabead experiments to a culture bag, while maintaining the same activation pattern seen in well plate studies, the transduction efficiency was consistently improved in cultures initiated with depleted starting material. The retroviral transduction protocol used in this study was reliant on interactions taking place at the surface of a culture vessel. A 100-mL culture bag possesses a lower volume-to-surface area ratio compared with a well plate, reducing the space for transduction to take place. This could mean that, in a well plate format, for some donors, any potential differences in transduction are obscured by optimal plate geometry. Interestingly, only one of the two donors (donor 7) that achieved increased transduction in the well plate geometry also demonstrated improved expansion as a result of CD14 depletion or T cell isolation. Donor 7 had the highest levels of CD14^+^ cells in the original leukapheresis material, accounting for 32% of the cell population, approximately 2 times higher than the other donors tested. It could be that the higher proportion of CD14 in the starting leukapheresis was more suppressive to manufacture , indicating that there may be a threshold as to when the percentage of CD14 become limiting to cell growth. This is supported by the fact that the CD19-depleted condition still presented lower transduction despite containing a similar percentage of CD3. Because of differences in their mechanism of infection, it should be noted that alternative transduction procedures, such as a lentivirus, may not be affected to the same degree as a retrovirus.

Although expansion was unaffected by starting material purity, there was some variability in the level of growth between donors under the same conditions. This is not unusual in CAR T cell production; for example, Brentjens et al.^37^ reported post-transduction fold expansions of 24 and 385 for two different individuals over a 16-day period. Interestingly, in well plate studies, TransAct achieved a higher level of expansion compared with Dynabeads.

As well as the effect of starting material on the manufacturing process, we also explored whether there were any changes in final CAR T cell product quality. Cytotoxicity analysis of the resulting CD3^+^CAR^+^T cells showed that those produced from purer starting materials outperformed those made from unsorted leukapheresis in terms of kinetics and reduction of the target cell population. Cytokine profile analysis after stimulation with Raji cells demonstrated that all CAR T cells were capable of expressing signals that trigger cell killing. Memory phenotyping of CAR T cells revealed that products stemming from an isolated CD3 population or monocyte-depleted starting material possessed, on average, 40% more T_N_ cells compared with those from unsorted leukapheresis. The ideal phenotypic profile for CAR T cells is widely debated; however, less differentiated CAR T cells have been shown to produce stronger anti-tumor efficacy as opposed to their more differentiated effector counterparts.^38^^,^^39^ The different compositions of phenotypes likely contribute to the cytotoxicity shown in Figure 6B, with the more naive product from depleted starting material outperforming the more differentiated product from unprocessed leukapheresis. Furthermore, in this study, CAR T cells produced from CD14-depleted material always produced the highest level of T_CM_ cells, which has been shown to correlate with persistence *in vivo*.^40^

Gene expression through RNA analysis (Figure 7C) showed that CAR T cells generated from starting material depleted of monocytes or isolated CD3^+^ cells expressed lower levels of genes associated with activation and cytotoxicity, such as chemokine receptor type 2 (CCR2), CCR5, Fas ligand (FASLG), and granzyme A (GZMA). As the cytotoxicity data had shown that CAR T cells manufactured from enriched starting materials outperformed those made from unsorted leukapheresis, this suppressed gene expression was not anticipated. However, RNA analysis was carried out prior to stimulation with an antigen, which means that downregulation of genes associated with activation/cytotoxicity is likely a result of cells being in a rested state. This raised the question of why the unsorted leukapheresis CAR T cells were not in the same state. Activation data (Figure 4A) revealed that unsorted leukapheresis contained a significantly larger population of CD25^−^CD69^+^ T cells compared with the other tested conditions. This suggests a delay in their activation state, resulting in higher expression of associated genes in the final product compared with the other conditions. This is supported by the Nanostring analysis, with genes associated with activation appearing to be upregulated in unsorted leukapheresis material compared with the other tested conditions (Figure 7D). A more rested and naive condition is a desirable characteristic for T cells because cells are ready to be re-stimulated, expanded rapidly, and differentiated into cytotoxic effectors.^41^^,^^42^ The more naive and rested state of the cells is likely to be the underlying cause of the differences in the cytotoxic action (Figure 6B). This is interesting because it demonstrates that starting material not only affects processing outputs but also fundamentally alters the final product. The genes shown in Figure 7C are only a snapshot of the genes that varied between the processed and unprocessed starting materials. In fact, CAR T cells produced from isolated CD3^+^ T cells were found to have 139 genes that were significantly up/downregulated compared with those produced from unsorted leukapheresis , whereas monocyte-depleted CAR T cells had 122 genes, indicating that, even though they have similar cytotoxicity, they are still fundamentally different from one another. Because of the small sample size in this study, additional donors are needed to further support these findings.

All experiments in this study were carried out using healthy donor material. Despite this, we have seen clear improvements in processing outcomes and changes in final product characteristics when activating with CD3/CD28 microbeads. Conversely, the TransAct nanomatrix activation reagent appears to be more able to handle variable starting materials. Inclusion of patient material in future experiments would be of interest because of its potentially lower quality and higher proportions of cell impurities, in particular the presence of blast cells^10^ and monocytes at higher levels.^4^^,^^20^^,^^43^ In summary, we have shown that the composition of starting material can affect the final product, altering phenotype profiles, genetic expression, and, ultimately, CAR T cell cytotoxicity. When using Dynabeads to activate cells, removal of CD14^+^ monocytes is a simple way to enhance processing efficiency without total enrichment of the CD3 population, potentially requiring only a simple pre-processing step in place of complex and expensive magnetic cell sorting.

## Materials and methods

### Culture preparation and activation

Peripheral blood leukapheresis products were obtained from the NHS or purchased as LeukoPaks from AllCells, Cellex, and Anthony Nolan as part of research study NREC ID 15/LO/1322.

Cryostored leukapheresis products from 6 donors were thawed rapidly in a water bath and washed with PBS (Sigma, Gillingham, UK). Leukapheresis products from each donor were purified using magnetic cell sorting techniques according to the manufacturer’s instructions (Miltenyi Biotec, Surrey, UK) to produce untouched populations depleted of CD19^+^ (130-050-301), CD14^+^ (130-050-201), or CD14^+^, CD15^+^, CD16^+^, CD19^+^, CD34^+^, CD36^+^, CD56^+^, CD123^+^, and CD235a^+^, producing untouched CD3^+^ cultures (130-096-535). Briefly, cells were pelleted and combined with antibody and antibody-conjugated magnetic beads before incubation at 2°C–8°C for 10–15 min. Cells were washed prior to loading onto LS columns mounted in a QuadroMACS separator, where selection was carried out. Columns were washed 3 times with PBS/EDTA + 0.5% human serum albumin (HSA) buffer, with unbound material collected and retained for processing. A biotin control was produced by following the steps for CD14 depletion, replacing CD14^+^ beads with a biotin-coated equivalent (130-090-485). The resulting populations were resuspended alongside unsorted leukapheresis products at 1 × 10^6^ cells/mL in RPMI culture medium supplemented with 10% heat-inactivated fetal bovine serum (FBS) and 1% Glutamax (Gibco, Thermo Fisher Scientific, Loughborough, UK) or TexMacs (Miltenyi Biotec, Surrey, UK) supplemented with 3% human serum (BioIVT, Sussex, UK). Cells were seeded into 100-mL differentiation bags (Miltenyi Biotec, Surrey, UK) or 24-well plates (Corning, NY, USA) and incubated overnight at 37°C and 5% CO_2_. Blood cells were harvested after overnight rest and activated relative to CD45^+^CD3^+^ content using Dynabeads (Thermo Fisher Scientific, Loughborough, UK) or TransAct (Miltenyi Biotec, Surrey, UK). Dynabeads were added at a 1:1 ratio to CD3^+^ cells in complete RPMI medium (supplemented medium + 300 IU/mL of IL-2 [Miltenyi Biotec, Surrey, UK]). TransAct was diluted 1:100 in complete TexMacs (supplemented medium + 300 IU/mL of IL-2 [Miltenyi Biotec, Surrey, UK]). Cells were transferred back into 100-mL differentiation bags or well plates at 500,000 or 1,000,000 T cells/mL, respectively, and incubated at 37°C and 5% CO_2_ for 48 h.

### Transduction and expansion

Differentiation bags (Miltenyi Biotec, Surrey, UK) or untreated well plates (Falcon, Corning, NY, USA) were coated with Retronectin (Takara Bio, Kusatsu, Japan) diluted in PBS to achieve a concentration of 1.72 mg/cm^2^ for 2 h at room temperature proceeded by overnight at 4°C. The coating solution was then removed from the culture vessels, and a retrovirus expressing GFP or a CAR (FMC63) was added. Vessels loaded with virus were spun at 1,000 × *g* for 40 min. After activation, cells were harvested from culture bags or vessels, and Dynabeads were removed. Cells were reseeded at 0.5 × 10^6^ cells/mL (culture bags) or 0.3 × 10^6^ cells/mL (well plates) in their respective complete culture medium. Cultures were incubated overnight at 37°C and then harvested, washed, and reseeded for expansion. Cultures were incubated for 72 h before being fed with complete medium and expanded for an additional 24 h. At the end of the culture bag experiments, samples were cryostored for future analysis.

### Flow cytometry

Unless otherwise stated, all antibodies and viability stains were from BD Biosciences (Wokingham, UK). Phenotype samples were taken before and after activation and at the end of expansion. Cells were stained with antibodies against CD45, CD3, CD14, CD19, CD56, CD34 (Biotechne, Abingdon, UK), CD4, CD8, and Live/Dead stain (7-Aminoactinomycin D, 7AAD). Activation samples were taken 48 h after reagent addition and stained with antibodies against CD3, CD4, CD8, CD25, CD45, and CD69. When following the GFP vector protocol, cell viability was identified using the Live/Dead stain 7AAD. For FMC63 vector studies, cells were stained with a fixable viability stain (BD Horizon FVS780), with cells fixed after staining with 2% paraformaldehyde (PFA). Memory phenotype was measured on thawed products by staining cells with CD3, CD34, CCR7, CD45RA, and 7AAD.

### Cytotoxicity assay and cytokine analysis

CD19^+^ SKOV3 cancer cells expressing RFP were co-cultured at a 1:1 ratio with CD3^+^CAR^+^ T cells and dispensed into a 96 well-plate (Corning, NY, USA). Plates were put into an IncuCyte S3 2018A plate reader (Sartorius, Hertfordshire, UK), and the red cell count was recorded every hour for 72 h. For cytokine release assays, Raji cells were combined at a 4:1 ratio with CD3^+^CAR^+^ T cells. Cell mixes were incubated for 48 h, and supernatants were retained and loaded into a customized Ella cartridge to analyze expression of IL-2, GZMB, IFNγ, and TNF-α using the Ella Simple Plex system (ProteinSimple, Biotechne, San Jose, CA, USA), operated according to the manufacturer’s instructions.

### Gene analysis

CAR T cells were purified by staining with CD34-APC^26^ (Biotechne, Abingdon, UK) antibody, followed by selection using anti-APC microbeads (Miltenyi Biotec, Surrey, UK). RNA was extracted from CAR T cells using the RNeasy Mini Kit (QIAGEN, Manchester, UK), following the manufacturer’s instructions. Samples were then prepared as outlined by the manufacturer for the Nanostring nCounter Sprint cartridge using the Nanostring CAR-T Characterization Panel (Nanostring, Amersham, UK). Analysis was carried out using Nanostring nSolver software, comparing the mRNA results from CARs produced from purified starting material with those produced from unsorted leukapheresis products.

### Statistical analysis

Statistical analyses were performed using analysis of variance (ANOVA). For multiple comparisons, Dunnett’s hypothesis testing was used, comparing means of purified material with those from unsorted leukapheresis products; the significance level was set at 0.05. Analysis was carried out using GraphPad Prism 8.

## Supplemental information

Document S1. Figure S1

Document S2. Article plus supplemental information
