# Supplementary material for: Enriching leukapheresis improves T cell activation and transduction efficiency during CAR T processing
Source: Mol Ther Methods Clin Dev. 2021 Feb 6;20:675–87. doi: 10.1016/j.omtm.2021.02.002 (PMC7917474; doi:10.1016/j.omtm.2021.02.002)
Supplement: Document S1. Figure S1 [file mmc1.pdf]

**OMTM, Volume *20***

## Supplemental information

# Enriching leukapheresis improves T cell activation and transduction efficiency during CAR T processing

**Elsa Noaks, Carlotta Peticone, Ekaterini Kotsopoulou, and Daniel G. Bracewell**

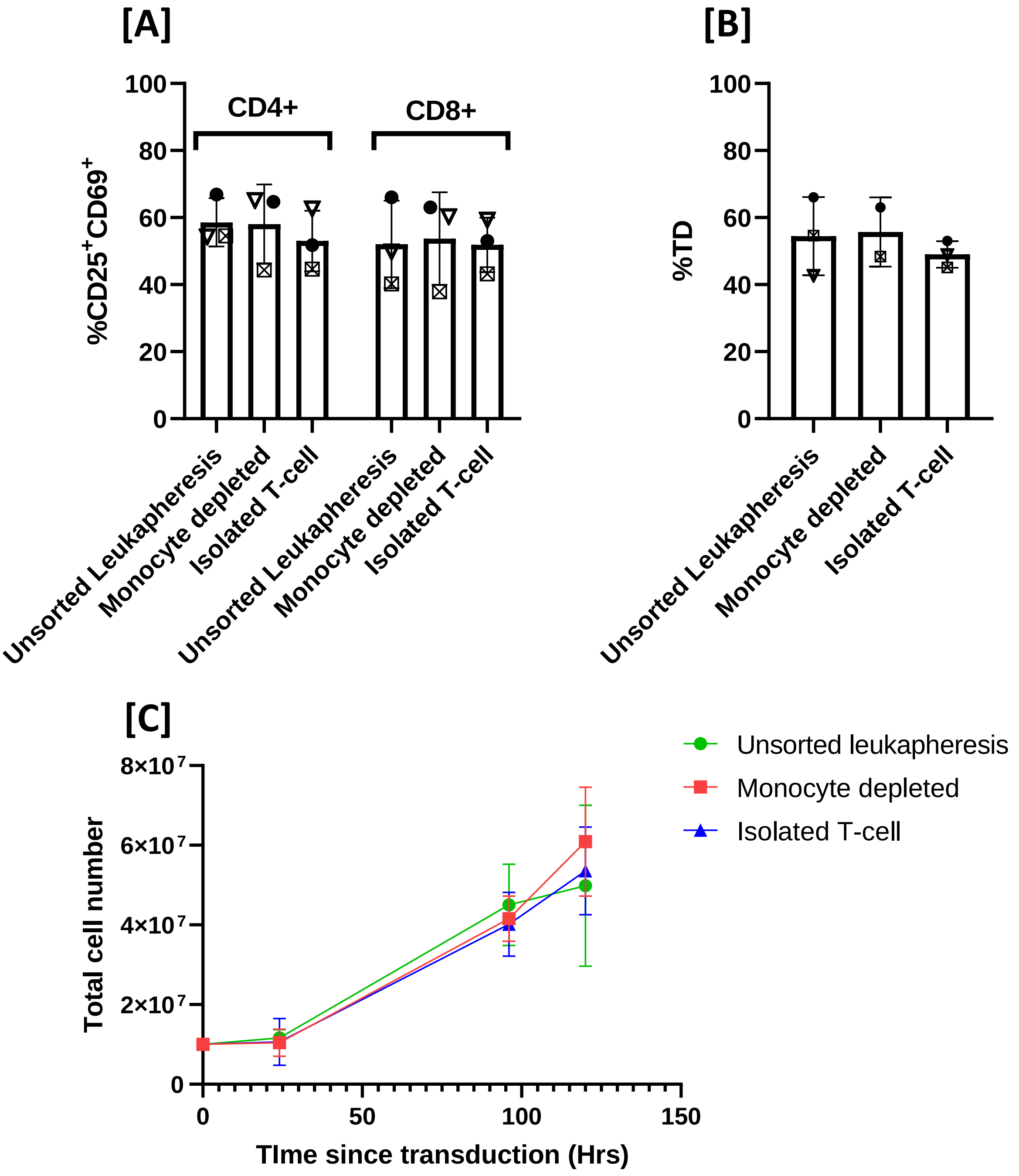


**Figure S1 – Results from TransAct based activation in differentiation bags** [A] CD25+CD69+ expression by CD4+/CD8+ T-cells subpopulations and [B] % Transduction efficiency (TD) for donor 1 (●), donor 10 (▽) and donor 11 (⊠) where donor 1 was transduced with the GFP vector and donor 10/11 with the CAR vector. [C] Growth curves after transduction (where 0hrs since transduction is equivalent to 48 hrs post activation) for unsorted leukapheresis, isolated T-cell and monocyte depleted conditions. Error bars represent the mean +/- 1 SD where bar height equals the mean.
